# Alternative patterns of sex chromosome differentiation in *Aedes aegypti* (L)

**DOI:** 10.1186/s12864-017-4348-4

**Published:** 2017-12-04

**Authors:** Corey L. Campbell, Laura B. Dickson, Saul Lozano-Fuentes, Punita Juneja, Francis M. Jiggins, William C. Black

**Affiliations:** 1 Department of Microbiology, Immunology and Pathology, Colorado State University, Campus Delivery 1692, Fort Collins, CO 80523 USA; 2 Department of Genetics, University of Cambridge, Downing Street, Cambridge, CB2 3EH UK

**Keywords:** Population genetics, Arbovirus vector, Dimorphic traits, Genomics, Evolution of reproductive proteins, Sex determination

## Abstract

**Background:**

Some populations of West African *Aedes aegypti*, the dengue and zika vector, are reproductively incompatible; our earlier study showed that divergence and rearrangements of genes on chromosome 1, which bears the sex locus (*M*), may be involved. We also previously described a proposed cryptic subspecies SenAae (PK10, Senegal) that had many more high inter-sex F_ST_ genes on chromosome 1 than did *Ae.aegypti aegypti* (Aaa, Pai Lom, Thailand). The current work more thoroughly explores the significance of those findings.

**Results:**

Intersex standardized variance (F_ST_) of single nucleotide polymorphisms (SNPs) was characterized from genomic exome capture libraries of both sexes in representative natural populations of Aaa and SenAae*.* Our goal was to identify SNPs that varied in frequency between males and females, and most were expected to occur on chromosome 1. Use of the assembled AaegL4 reference alleviated the previous problem of unmapped genes. Because the *M* locus gene *nix* was not captured and not present in AaegL4, the male-determining locus, per se, was not explored. Sex-associated genes were those with F_ST_ values ≥ 0.100 and/or with increased expected heterozygosity (*H*_*exp*_, one-sided T-test, *p* < 0.05) in males. There were 85 genes common to both collections with high inter-sex F_ST_ values; all genes but one were located on chromosome 1. Aaa showed the expected cluster of high inter-sex F_ST_ genes proximal to the *M* locus, whereas SenAae had inter-sex F_ST_ genes along the length of chromosome 1. In addition, the Aaa *M*-locus proximal region showed increased *H*_*exp*_ levels in males, whereas SenAae did not. In SenAae, chromosomal rearrangements and subsequent suppressed recombination may have accelerated X-Y differentiation.

**Conclusions:**

The evidence presented here is consistent with differential evolution of proto-Y chromosomes in Aaa and SenAae.

**Electronic supplementary material:**

The online version of this article (doi: 10.1186/s12864-017-4348-4) contains supplementary material, which is available to authorized users.

## Background

The dengue, yellow fever, chikungunya and zika vector, *Aedes aegypti,* has at least two major subspecies in tropical and subtropical regions; these consist principally of forest and peridomestic types [1–3]. Although morphological features such as abdominal scale patterns have been used to differentiate these groups, definitive molecular markers for subspecies identification are not yet available [1, 2, 4, 5]. Population-specific differences in west African population vector competence for flaviviruses have been described [6, 7]; and a trend toward reproductive isolation [8] may contribute toward these differences, as well as other traits [6, 7, 9]*. Ae. aegypti* has a dominant male-determining sex locus (*M*) on chromosome 1, for which males are heterozygous (*Mm*). This locus is primarily responsible for sex determination [10], however male and female chromosomes are also cytologically distinct [11]. The male-determining factor (M factor) *nix*, an M-linked myosin heavy chain gene, *myo-sex*, and two sex determination transcription factors have been characterized [10, 12–15], but little else is known about the specific genes contributing dimorphic phenotypes in aedine mosquitoes.

Metazoan proteins involved in mating and reproduction evolve more rapidly than genes in other functional groups, and this phenomenon may contribute to reproductive isolation and subsequent speciation (reviewed in [16–18]). The opposing evolutionary forces of male sexual selection and female conflict may be involved in this process [19, 20]. Rapid sex-associated gene evolution has been described in *Anopheles* mosquitoes [21] and drosophilids [22]. Haerty et al. showed rapid divergence of sex-associated genes in drosophilid males [22]. Such rapid evolution is also supported in taxa without a hemizygous X, as is the case in *Ae. aegypti* [8, 23], and has been attributed to sexual selection acting mostly on males [24]. It is expected that alleles with sexually antagonistic effects on fitness would accumulate on sex chromosomes, where they would be expressed predominantly or exclusively in the sex where they are advantageous (reviewed in [25]). In a species, such as *Ae. aegypti,* with recombining homomorphic sex chromosomes, these genes are expected to be enriched in regions tightly linked to the *M* locus. Because recombination should be suppressed in the *M* locus proximal region, differentiation of males and females likely occurs by genetic drift or possibly by selection of specific genes. For these reasons, analysis of sex-specific genetic variation in reproductively isolated mosquito populations could reveal gene diversity contributing to reproductive isolation and speciation [26].

A Senegalese sylvatic population (PK10, SenAae) has increased genetic and structural diversity at chromosome 1 compared to the type form *Ae. aegypti aegypti* (Aaa), possibly due to chromosomal rearrangements [26, 27]. In addition, PK10 showed reproductive incompatibility when mated to PK10 males with different abdominal banding patterns [26]. Interestingly, this strain also lacked the expected genetic linkage of the *white-eye* and the *M* locus in 26% of genetic families [27], which was consistent with the observations of sex chromosome structural diversity. Further, high throughput sequencing (HTS) showed that overall standardized variance (F_ST_) was greater in SenAae than the representative type form, Aaa [27]. These unusual attributes in SenAae sex chromosomal structure and reproductive isolation led us to further explore sex-specific genomic polymorphisms in order to increase understanding of sex-specific differences in *Aedes* subspecies.

Therefore the over-arching goal of this study was to extend our earlier study [27] and use population genomics analyses of SenAae and Aaa to characterize sex-specific allele frequency differences. Our hypothesis was that genes with high sex-specific or inter-sex F_ST_ values would be located proximal to the *M* locus on chromosome 1 [28]. We used orthology information to predict whether these genes would be involved in in sex determination, reproduction and/or sexual dimorphic traits. Exome capture [29] genomic DNA (gDNA) HTS data from independent replicate pools (*n* = 12) of adult *Ae.aegypti* males and females were compared for two geographically and genetically distinct populations, with subsequent analysis of sex-specific single nucleotide polymorphisms (SNPs). The collections, SenAae and the type form Aaa from Thailand, have been highlighted in previous studies [26, 27, 30]. Standardized variance in SNP frequencies (F_ST_) was used to compare sex-specific differences [31]. Thus, in the context of this work, high inter-sex F_ST_ values revealed SNPs that differed in frequency between males and females. We also expected that genes linked to the *M* locus would be more heterozygous in males [8, 32]. The Hardy-Weinberg expected heterozygosity (*H*_*exp*_) score indicated the predicted level of sex-specific genetic diversity. Genes with high *H*_*exp*_ and/or F_ST_ levels may play roles in mating, sex determination, dimorphic development or trends in reproductive isolation.

## Results

### Exome-wide analysis of sex-specific polymorphisms

Exome-captured HTS libraries were sequenced from pools of Aaa and SenAae males and females; Table 1 shows library-specific information and overall polymorphism statistics. Two biological replicates for each pool (12 mosquitoes per pool) of males and females from each location produced a total of eight libraries (SenAae: 2 male PK10, 2 female PK10; Aaa: 2 male Thai, 2 female Thai). Roughly 34-38 million trimmed reads were produced from SenAae, and 18-25 million reads were obtained from the Aaa collection (Additional file 1). The chromosome-length assembled Aaa genome was used as a reference for all alignments (AaegL4) [33–35]; and 90-92% of trimmed reads aligned in each population (Additional file 1). Sex-specific polymorphisms were identified at each nucleotide site (at least 15 read counts per site) using the F_ST_ calculation (see Methods); sex-specific *H*_*exp*_ scores were also calculated [31]. SNPs that were completely fixed for both sexes but different from the reference, also known as monomorphic SNPs, were removed. The Aaa collection had about 1.9 million sex-specific polymorphisms and SenAae had about 3.0 million (Table 1). To rule out the possibility that population-specific differences arose from dissimilarities in sequencing coverage, the ratio of variant sites (_*_ 1000) per aligned nucleotide were calculated on each chromosome (Additional file 1). In SenAae, the variant/aligned ratio ranged from 2.0-3.6 per chromosome, while in Aaa, they ranged from 2.6-4.8. Therefore, the overall relative number of variants per aligned nucleotide in Aaa was higher than that of SenAae, indicating that the features described below were not due to library size differences.

**Table 1:** Polymorphisms and Coverage

	Aaa		SenAae	
Monomorphic SNPs -Excluded	21,849,618		23,861,997	
Number of variant sites	1,901,845		3,044,292	
Coverage per nucleotide^a^-	-Min	60		60
	-Max	3564		3745
	-Mean	180		261.4
	-Median	152		214
Allele frequency Statistics	Aaa		SenAae	
	Female	Male	Female	Male
H_exp_ across all genes				
Mean (95% cI)	0.097 +/− 0.084	0.098 +/− 0.084	0.113 +/− 0.090	0.112 +/− 0.089
Median	0.024	0.024	0.029	0.026
Mean sample variance	0.002	0.002	0.002	0.002
Standard deviation	0.043	0.043	0.046	0.046
Chr 1 H_exp_				
Mean (95% cI)	0.099 +/− 0.084	0.102 +/− 0.086	0.112 +/− 0.089	0.111 +/− 0.089
Median	0.025	0.027	0.031	0.027
Mean sample variance	0.002	0.002	0.002	0.002
Standard deviation	0.043	0.044	0.046	0.045
Increased male H_exp_, T-test *p* value		2.20E-16		ns
M locus proximal region				
Mean (95% cI)	0.074 +/− 0.074	0.082 +/- 0.078	0.082 +/− 0.078	0.081 +/− 0.034
Median	0.018	0.02	0.016	0.015
Mean sample variance	0.001	0.002	0.002	0.0003
Standard deviation	0.038	0.04	0.04	0.0176
Increased male H_exp_, T-test *p* value		2.20E-16		ns

*ns* not significant (one-sided T test, *p* < 0.05)

^a^Collection-wide total coverage

**Fig. 1:**
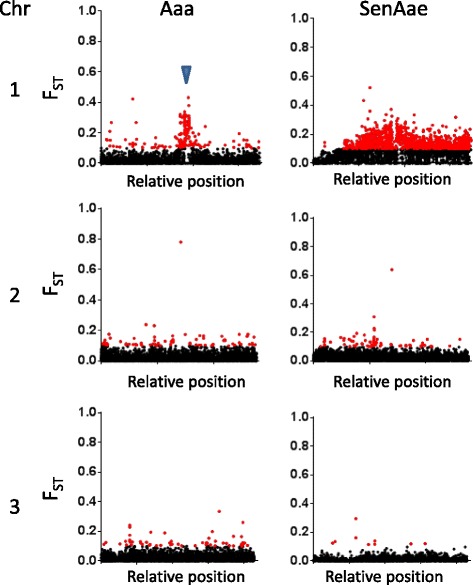
Inter-sex F_ST_ values vary among *A.aegypti* populations. Relative position of gene-wise F_ST_ values per chromosome. Red dots indicate genes with F_ST_ values ≥ 0.100 (Aaa, (Thai) collection, *n* = 304; SenAae (PK10) *n* = 1310); black dots indicate F_ST_ values below the threshold. Blue carat indicates predicted location of *nix* at the *M* locus

Polymorphisms were examined to identify gene-wise inter-sex F_ST_ values (Fig. 1, Materials and Methods), which were expected to follow a *beta* distribution (Additional file 2). We chose a cut-off of F_ST_ ≥ 0.100 to identify genes of interest for this study. This cut-off was chosen rather than a percent cut-off, such as the upper 5%, because Aaa showed many fewer genes with F_ST_ greater than 0.100 in the upper 5% than did SenAae. For example, in the upper 5% subset, Aaa had 441 genes with F_ST_ < 0.100, while SenAae had none. The chromosome-length reference allowed us to examine inter-sex F_ST_ averages per gene relative to each physical location. The number of high inter-sex genes was significantly higher on chromosome 1 than either chromosome 2 or 3 (Fisher’s Exact test, SenAae, *p* < 0.0001, Aaa, *p* < 0.0001). Interestingly, Aaa had a distinct cluster of high inter-sex F_ST_ genes on chromosome 1, with a few in distal locations (*n* = 171). This region overlaps a similar region of high inter-sex F_ST_ reported for the Liverpool Aaa strain [28]. This is consistent with retention of the sex locus in Aaa, with a cluster of inter-sex F_ST_ genes proximal to *nix* (Fig. 1). Importantly, the male-specific *M* locus *nix* was not included in the AaegL4 reference or in our capture probes [12, 13, 36], however, predictions from AaegL4 indicate that *nix* is located between AAEL015064-RA and AAEL014760-RA at the location of the Aaa high F_ST_ cluster [35].

SenAae had high inter-sex F_ST_ genes across most of the chromosome (*n* = 1233). This pattern is consistent with extensive chromosome length X-Y differentiation, which is different from findings of other aedine populations [28]. The high level of reported SenAae genetic diversity may have contributed to the chromosome-wide pattern [26, 27], as mosquito pools rather than individuals were evaluated in this study.

**Fig. 2:**
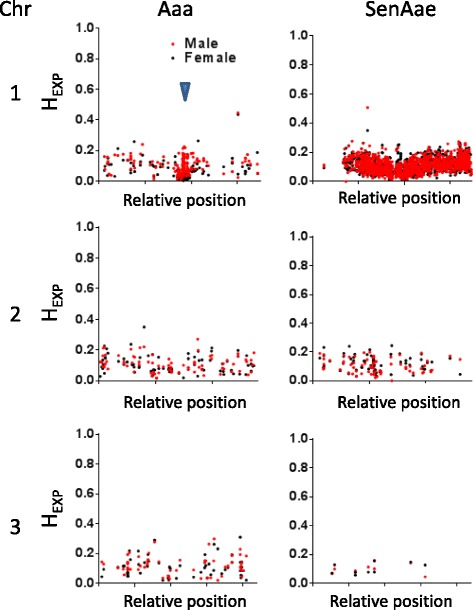
Inter-sex H_ex*p*_ values vary among *A.aegypti* populations. Relative position of gene-wise H_exp_ values for those genes in the high F_ST_ group (F_ST_ ≥ 0.100). Red dots indicate male gene-wise H_exp_ values; black dots indicate female gene-wise H_exp_ values. Blue carat indicates predicted location of *nix* at the *M* locus

Organisms with a single sex-determining locus, such as *Aedes spp*., would be expected to bear sexually dimorphic heterozygosity proximal to the sex locus, and males should have greater heterozygosity at these sites. We assessed H_exp_ values along the length of chromosome 1, testing specifically for higher average H_exp_ values in males over females (one-sided T-test). Along the entire length of chromosome 1, Aaa males had increased heterozygosity levels; this was especially marked in the central third of chromosome 1 (Fig. 2 and Table 1, one-sided T-test, *p* < 2.2E-16), which also corresponds to an area of reduced recombination reported by Fontaine et al. [28]. Curiously, increased male heterozygosity was not observed in any portion of Chromosome 1 in SenAae.

Though high inter-sex F_ST_ genes were expected only on chromosome 1, genes with association to sex were present on all three chromosomes in both populations. High inter-sex F_ST_ genes on chromosomes 2 and 3 could be involved in processes other than sex determination, such as reproduction, sexual dimorphic development or behavior. Alternatively, this category could also include genes that contribute to sex distortion phenotypes [37–39].

We expected that F_ST_ calculations of female-vs-female comparisons from each population should be reduced proximal to the *M* locus. Indeed, graphs of female-vs-female and male-vs-male comparisons indicate a marked reduction in F_ST_ values proximal to the M locus in females but not in males (Additional file 3). The high number of F_ST_ values > 0.100 shows the high level of differentiation between Aaa and SenAae.

### Common features of X-Y differentiation

**Fig. 3:**
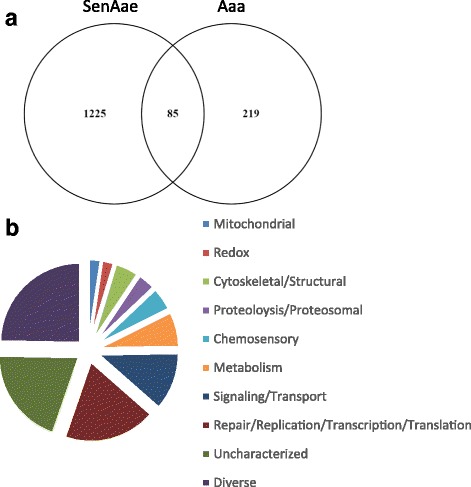
Functional categories of genes showing sex-specific polymorphisms. **a** The intersection of genes among both populations with high inter-sex F_ST_ values (≥ 0.100). **b** The resulting 85 common genes were classified by functional category and are shown as a portion of the pie chart. Legend: the list of functional groups, arranged from top to bottom, is represented in the pie chart clock-wise, starting at the top-most slice

Our gene-by-gene F_ST_ calculations provided a unique opportunity to explore specific high inter-sex F_ST_ genes that were shared among the two populations. The premise of this line of inquiry was to identify specific genes that may contribute to male-female differentiation. Indeed, a study of humans showed that high F_ST_ genes were enriched on X chromosomes relative to autosomes [40]. The intersection of high inter-sex F_ST_ genes (≥ 0.100, *n* = 85) among Aaa and SenAae was assessed (Fig. 3a, Additional file 4). As expected, chromosome 1 was most represented in this subset (Fisher’s Exact test, *p* < 0.001); just a single gene (AAEL001298) on chromosome 2 was present. The data are consistent with an overall lack of common autosomal high inter-sex F_ST_ genes, indicating that most autosomal high F_ST_ genes are due to population-specific trends. Importantly, high F_ST_ values on X-Y chromosomes could be due to genetic drift or sex-specific selection, therefore both are possible explanations for these high F_ST_ values [41]. Nevertheless, coordinated cis-regulation of gene expression on sex chromosomes has also been described [42] and provides support for the hypothesis that sex differentiation genes, other than the *M* locus, are present within the high F_ST_ clusters.

To further explore the common gene set, the genes were assigned to functional categories by orthology (BLAST, E^−20^ cut-off) to other dipterans [43] (Fig. 3b, Additional file 4). Excluding the diverse and uncharacterized subsets, the largest subset contained genes involved in DNA repair/replication/transcription/translation, which accounted for 18% of the total and was over-represented in this subset (hypergeometric analysis, *p* < 0.003). Nine genes had domains consistent with transcriptional activation or suppression activities. Sex-linked genes could also be those that contribute to sexually dimorphic phenotypes. For example, genes predicted to be involved in the chemosensory response (3.8%) were also represented, though not significantly over-represented. A possible sexual dimorphic bias in chemosensory function was expected, given that males and females have distinct food sources and mating behaviors [44, 45].

In insects, sex determination mechanisms are highly variable across genera and species, and sometimes vary within a single species, as occurs in *Musca domestica* [46–48]. One common mechanism of dipteran sex determination, which also holds true for *Ae.aegypti* [14, 15], is dimorphic regulation of alternative RNA splicing mechanisms (reviewed in [49]). Just two RNA processing/splicing or sex determination genes were in the high F_ST_ group common to both SenAae and Aaa populations, AAEL017421 and AAEL006713. Although a specific function for nucleolar protein 56 (AAEL017421) has not been identified, other nucleolar proteins are important for tissue-specific development and maintenance of heterochromatin and ribosomal RNA [50]. In addition, a U2 snRNP auxiliary factor subunit (AAEL006713) was also in this group. U-type snRNPs make up the canonical RNA-splicing machinery (reviewed in [49]).

Each collection was further interrogated to identify male seminal fluid genes [51]. 2 genes were common to both populations; they code for seminal fluid proteins AAEL010935, a gamma glutamyl transpeptidase, and AAEL014053, a vacuolar ATPase. In SenAae alone, AAEL003746, a gene with predicted 4-hydroxybutyrate CoA-transferase activity, and AAEL005790, a predicted malate dehydrogenase were identified. Also, in Aaa alone, AAEL008489, a predicted calcium ion binding protein, was identified.

## Discussion

Proto-Y chromosomes evolve from autosomes upon the acquisition of a male-determining factor (reviewed in [52, 53]). The evidence presented here is consistent with differential evolution of proto-Y chromosomes in Aaa and SenAae. We showed that Aaa had a cluster of high inter-sex F_ST_ genes (F_ST_ > 0.100) proximal to the *M* locus (Fig. 1). In contrast, SenAae showed high inter-sex F_ST_ along the majority of chromosome 1. In Aaa but not SenAae, the *M* locus proximal region had significantly higher male *H*_*exp*_ levels (Fig. 2), which is consistent with reduced recombination. Suppressed recombination is a necessary prelude to the development of heteromorphic sex chromosomes. The reason for the absence of these features in SenAae is unknown. It could be due to a high level of genetic diversity in this population but is also consistent with accelerated X-Y differentiation. For example, chromosomal rearrangements in SenAae [26] could have contributed to accelerated X-Y differentiation of chromosome 1. Indeed, chromosomal inversions can also reduce recombination rates in proto-sex chromosomes [54].

Both groups showed population-specific trends for high inter-sex F_ST_ genes on chromosomes 2 and 3. The identification of high intersex F_ST_ genes on autosomal chromosomes is consistent with previous studies that identified multiple independent loci contributing to the sex phenotype in other culicine species [55]. In addition, it is also consistent with the presence of high F_ST_ autosomal genes in flies that arose from sex-specific selection due to sexual-antagonistic mechanisms [41]. Alternatively, these could also be due to sex distortion trends, though we were unable to test this hypothesis in this study. In an organism heterozygous for a given pair of alleles, we expect equal recovery of each allele in the gametes. Loci in which this fails to occur constitute “meiotic drive” (MD) or “segregation distortion” systems. Because of the ease of detection, sex ratio distortion has been the best-studied system. In *Aedes aegypti*, [10] the male parent determines the sex ratio in progeny and, given normal segregation, equal numbers of males and females should occur. However, departures from a 1:1 sex ratio are often observed in culicine mosquitoes and have been best studied in *Aedes aegypti,* wherein 35 to 45% females are found in field collected populations [56]. A study of sex ratio in 19 laboratory strains revealed that some strains had ~50% females, others had a slight excess of males (~40% females) and a few showed distinct deviations in sex ratio (< 30% female) [57]. In 1976, a meiotic drive (MD) gene product that is tightly linked to and acts *in trans* with the *M* allele was observed to cause breakage of the *m* allele (female)-carrying chromosome [58]. It was proposed that the *m* allele carrying chromosome is sensitive (*ms*) or insensitive (*mi*) to MD. Additionally, some *m* alleles vary in their sensitivity to distortion over a range of haplotypes [37, 59–62].

Most recently, investigators selected for a strain in which only 14.7% of progeny are females [63]. This distortion is due to an inherited factor that causes a predominance of males in certain strains and for the progeny of single pair matings. The factor is transmitted only by males [64]. Several modifiers of MD have been identified. The *tolerance of Distorter* locus is near the *re* locus at 47 cM on chromosome 1 and results in a reduction in sex ratio distortion [39]. Another suppressor of MD is linked with the spot abdomen (*s*) locus on 29 cM on chromosome 2, and an enhancer of MD was linked with the black tarsus (*blt*) locus at 28 cM on chromosome 3 [65]. The actual genes associated with these genetic loci have not been identified.

## Conclusion

In Aaa, increased male heterozygosity levels and high intersex F_ST_ genes are consistent with the presence of a proto-Y chromosome (reviewed in [25]). In contrast, chromosomal rearrangements and subsequent suppressed recombination in SenAae may have accelerated X-Y differentiation, as the features observed in Aaa were absent. Our approach also allowed us to identify additional genes associated with sex, which may include candidates for *M* locus modifiers. However, further characterization will be required to confirm possible mechanisms. Taken together, these data could inform transgenic strategies for vector control and the overall understanding of evolution of sex-associated genes in aedine mosquitoes.

## Methods

### Samples and sequencing

SenAae (PK10) and Aaa (Thai) collections were processed as follows. Deep sequencing libraries were made from pools of F1 individuals collected from the PK10 forest, Senegal in 2011 and *Ae. aegypti aegypti* from a collection in Pai Lom, Thailand in 2002 [7, 66, 67]. For both comparisons, mosquitoes were collected as larvae, reared to adulthood and frozen until DNA extractions. Two biological replicates for each pool (12 mosquitoes per pool) of males and females from each location produced a total of eight libraries (2 male PK10, 2 female PK10, 2 male Thai, 2 female Thai). Prior to pooling, DNA in individual mosquitoes was quantified using Pico Green (Life Technologies, Thermo Fisher Scientific Inc.) and equal amounts of DNA per mosquito were pooled. A Covaris S2 sonicator (Covaris Ltd., Brighton UK) sheared pooled DNA to an average size of 500 bp. Sonication conditions were: duty cycle 10%, Intensity 5.0, Cycles per burst 200, Duration 40 s, Mode Frequency sweeping, Displayed Power 23 W, Temperature 5.5° to 6 °C. Each TruSeq DNA LT (v.2) library was prepared using 1 μg of sheared genomic DNA following manufacturer’s recommendations. Equimolar quantities of prepared libraries were pooled and enriched for coding sequences by exome capture using custom SeqCap EZ Developer probes (Nimblegen) [29]. In total, 26.7 Mb of the genome (2%) was targeted for enrichment, as described elsewhere [29]. Overlapping probes covering the protein coding sequence (not including UTRs) in the AaegL1.3 gene annotations (https://www.vectorbase.org/organisms/aedes-aegypti/liverpool-lvp/AaegL1.3) were produced by Nimblegen. Enrichment followed the Nimblegen SeqCap EZ protocol. Briefly, pooled TruSeq libraries were hybridized to the probes for 64 h, unbound DNA was washed away, and the targeted DNA was eluted and amplified. These were then sequenced on 2 lanes of a HiSeq2000 (Illumina) for paired-end 2 × 100 nt sequencing. TruSeq library preparation, exome capture and sequencing were performed by the High-Throughput Genomics Group at the Wellcome Trust Centre for Human Genetics (Oxford, UK) and produced reads with quality scores > 30.

### Bioinformatics

#### Alignments and population genetics pipeline

All raw reads were trimmed of adapters and filtered using cutadapt (v. 1.14) [68]. The AaegL4 genome build [35] of 18,769 transcripts was used, including all 5’UTRs, exons, introns, 3’UTRs. The 5′ and 3′ non-transcribed regions in previously reported alignments were excluded [27]. Individual replicate fastq files were aligned to the AaegL4 genome using GSNAP (version 2017-02-25), allowing 10% divergence [69]. Using SAMtools “mpileup” command [70], GSNAP outputs were converted to *.mpileup files. The “readcounts” command in Varscan2 (v2.3.5) [71] was used to convert *.mpileup files to readcounts output, using the following options: --min-coverage 15 --min-base-qual 30. The readcounts output listed each SNP as a single row and A, C, G, T, in/del in columns.

To address possible sequencing errors, the following steps were taken: 1) a minimum of 15 variants per SNP site were required for a site to be considered; 2) only reads with Q30 passed trimming (cutadapt); this quality score was also required at each base upon alignment to the reference; 3) only those SNP sites that were present in both replicate libraries were included in F_ST_ calculations. PCR duplicates were not removed, because of the evidence that removal does not significantly alter variant calls [72].

For each SNP, in-house FORTRAN (F77) scripts (available on request) used the variant coverage per SNP site to calculate the Fumagalli F_ST_. Between-group component (*a*_*s*_), a within-group component (*b*_*s*_) and F_ST_ calculated from *a*_*s*_ and *b*_*s*_ following Fumagalli [31] where:

$$ {a}_s=\frac{4{n}_i{\left({\widehat{p}}_{\left(i,s\right)}-{\widehat{p}}_s\right)}^2+4{n}_j{\left({\widehat{p}}_{\left(j,s\right)}-{\widehat{p}}_s\right)}^2-{b}_s}{2\left(2{n}_i{n}_j/\left({n}_i+{n}_j\right)\right)} $$

and $$ {b}_s=\frac{n_i{\alpha}_{\left(i,s\right)}+{n}_j{\alpha}_{\left(j,s\right)}}{n_i+{n}_j-1} $$

where $$ {\alpha}_{\left(i,s\right)}=2{\widehat{p}}_{\left(i,s\right)}\left(1-{\widehat{p}}_{\left(i,s\right)}\right) $$ and $$ {\alpha}_{\left(j,s\right)}=2{\widehat{p}}_{\left(j,s\right)}\left(1-{\widehat{p}}_{\left(j,s\right)}\right) $$.

${\widehat{p}}_{\left(i,s\right)}$ is the coverage of a nucleotide at SNP site (*s*) divided by the total coverage of *s* in collection (*i*). *n*_*i*_ and *n*_*j*_ are the number of mosquitoes sampled in collections *i* and *j*, and ${\widehat{p}}_s$ is the coverage of a nucleotide at *s* in both *i* and *j* collections divided by the total coverage of *s* in both *i* and *j* collections. The estimate of F_ST_ for *s* is:

$$ {F}_{ST}(s)=\frac{a_s}{a_s+{b}_s} $$

and for an entire gene (*g*) with *m* SNPs is:

$$ {F}_{ST}(g)=\frac{\sum \limits_{s=1}^m{a}_s}{\sum \limits_{s=1}^m\left({a}_s+{b}_s\right)} $$

Genes were annotated using Gene Ontology terms and SwissProt functional annotation data listed in AegyXcel (http://exon.niaid.nih.gov/transcriptome.html#aegyxcel), using a cut-off e value of E^-20^.

Hardy-Weinberg expected heterozygosity (*H*_*exp*_) values were calculated for SNP sites that were present in both males and females using the following formula, ${\alpha}_{\left(i,s\right)}=2{\widehat{p}}_{\left(i,s\right)}\left(1-{\widehat{p}}_{\left(i,s\right)}\right)$; *α*_(*i*, *s*)_ is expected heterozygosity (*H*_*exp*_). ${\widehat{p}}_{\left(i,s\right)}$ is the coverage of a variant at a SNP(*s*) site divided by the total coverage of *s* in the collection (*i*).

### Female-vs-female and male-vs-male comparisons

Similar to the inter-sex comparisons, F_ST_ was also calculated for replicate female SenAae and Aaa libraries (Pk10 female vs Thai female). F_ST_ was also calculated for male-vs-male libraries to obtain the plots shown in Additional file 3.

### Statistics

Descriptive statistics were calculated in R (version 3.0.2). We evaluated H_ex*p*_ values along the length of chromosome 1 using a one-sided T-test (*p value* < 0.05) that tested specifically for higher average H_exp_ values in males over females. The ratio of variant sites per nucleotide of aligned reads was calculated as follows. Using flagstat (SAMtools), the number of aligned reads was determined in the reads aligned to reference *.bam files, and multiplied by the read-length (100nts) to achieve the total nucleotides aligned. The number of variants per chromosome was multiplied by 1000 and divided by the estimated total nucleotides aligned (Additional file 1).

Studies of *Ae. aegypti* RAD-tag and SNP-CHIP analyses allowed just 2 alternate alleles per locus [73, 74], whereas, here, loci with 3 or more alternate alleles were included in the final analysis. Moreover, our approach is not subject to ascertainment bias, as occurs when a small number of SNPs from the entire genome are analyzed [75, 76]. This systematic bias occurs when limited loci are analyzed rather than complete genotypic profiles.

## Additional files

Additional file 1: Sequencing Statistics. SenAae (PK10) and Aaa (Thai) HTS details. Total trimmed reads aligned to the AaegL4 reference; percent reads mapped; percent properly paired; number of variant sites; ratio of variant sites per aligned nucleotide*1000. (XLSX 16 kb)

Additional file 2: F_ST_ frequency distributions. SenAae (PK10) and Aaa (Thai). (PDF 19 kb)

Additional file 3: F_ST_ frequency distributions for female-vs-female and male-vs-male comparisons. SenAae (PK10) and Aaa (Thai). Black dots indicate average F_ST_ values < 0.100; red dots indicate FST values ≥ 0.100. (PDF 699 kb)

Additional file 4: Genes with significant sex-association values common to both populations. Vectorbase number (VBN), transcript, Chr, function, Function_description_Vectorbase. (XLSX 166 kb)

## Abbreviations

Aaa: *Aedes aegypti aegypti*
AaegL4: Chromosome-length *Ae. aegypti aegypti* reference sequence
*blt*: *black tarsus* locus
DNA: Deoxyribonucleic acid
F_ST_: Standardized variance, a measure of genetic association
*H*_*exp*_: Expected heterozygosity
*m* allele: Female recessive locus
*M* locus: Male determining locus
MD: Meiotic drive
PK10: *Aedes aegypti* collection from southeast Senegal
*s*: *Spot* locus
SenAae: Senegal *Aedes aegypti* collection
SNP: Single-nucleotide polymorphisms
