# Supplementary figures and images for: Alternative patterns of sex chromosome differentiation in Aedes aegypti (L)
Source: BMC Genomics. 2017 Dec 4;18:943. doi: 10.1186/s12864-017-4348-4 (PMC5716240; doi:10.1186/s12864-017-4348-4)

**Thai frequency distribution**

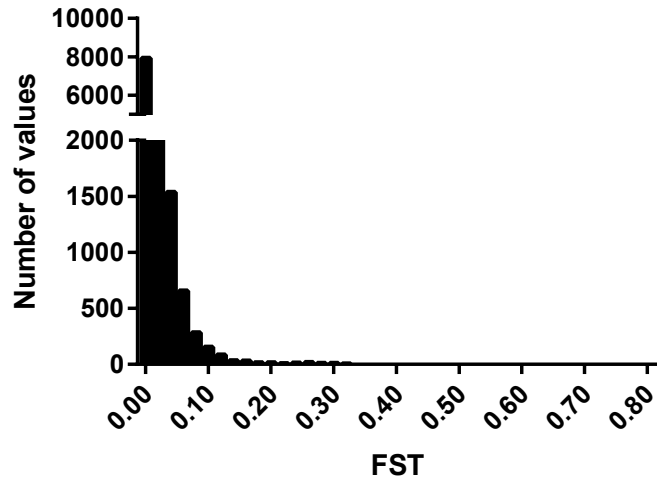

**PK10 frequency distribution**

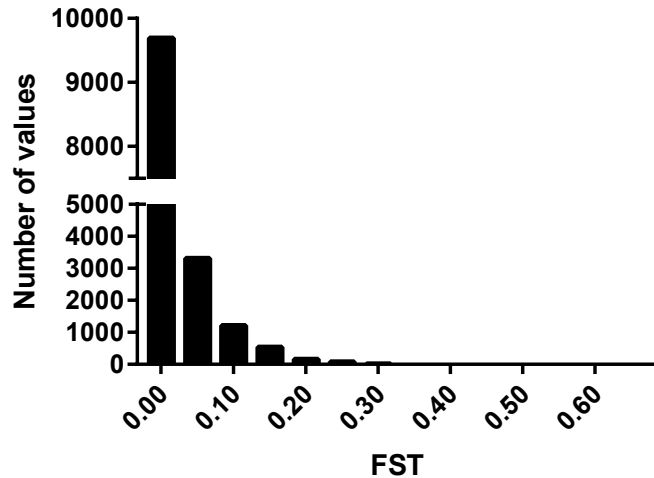

Supplement: Supplementary file 2 — FST frequency distributions. SenAae (PK10) and Aaa (Thai). (PDF 19 kb) [file 12864_2017_4348_MOESM2_ESM.pdf]

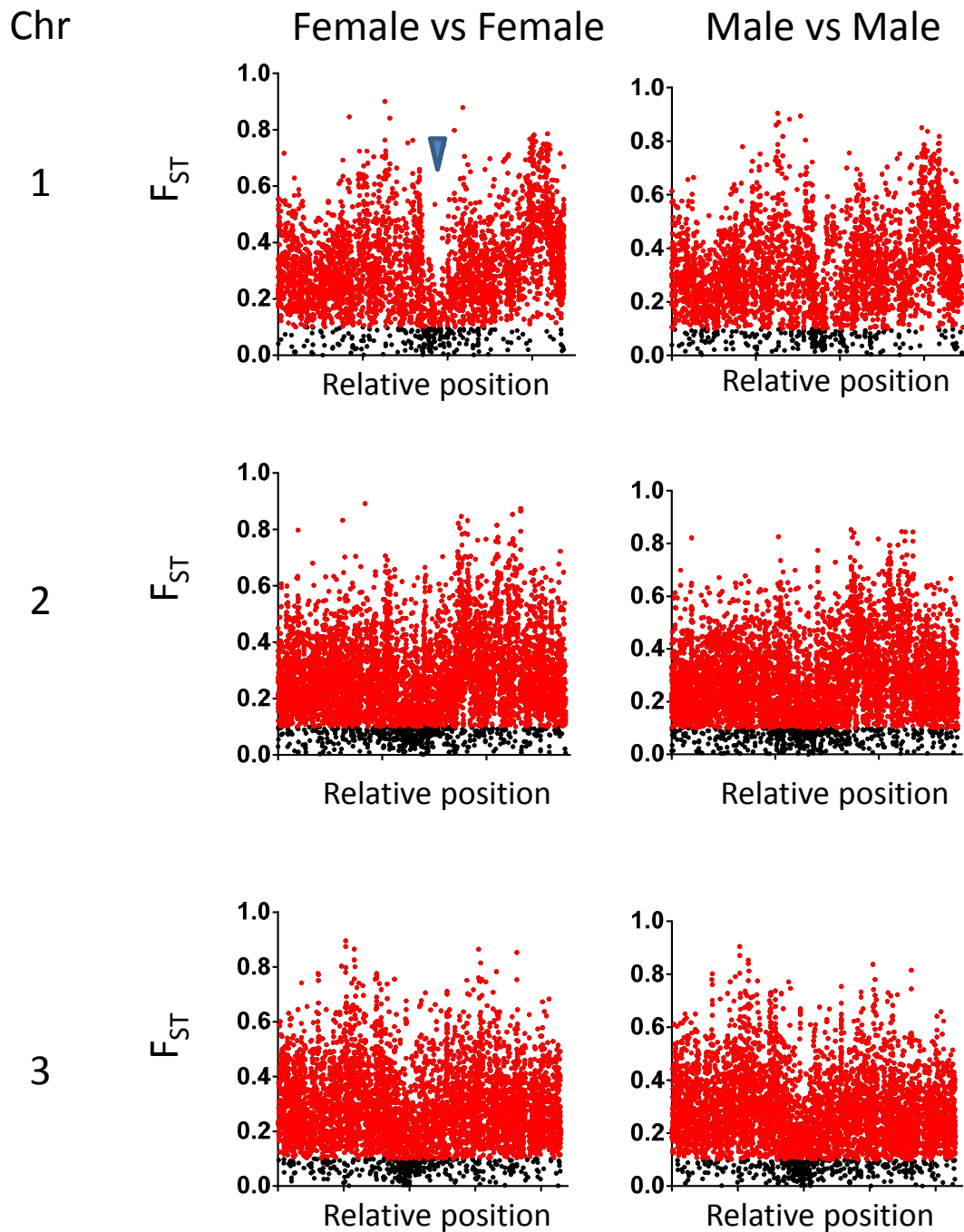

Supplement: Supplementary file 3 — FST frequency distributions for female-vs-female and male-vs-male comparisons. SenAae (PK10) and Aaa (Thai). Black dots indicate average FST values < 0.100; red dots indicate FST values ≥ 0.100. (PDF 699 kb) [file 12864_2017_4348_MOESM3_ESM.pdf]
